# m^6^A-modified circFNDC3B inhibits colorectal cancer stemness and metastasis via RNF41-dependent ASB6 degradation

**DOI:** 10.1038/s41419-022-05451-y

**Published:** 2022-11-29

**Authors:** Wei Zeng, Jin-Feng Zhu, Jian Guo, Gen-Jie Huang, Li-Sha Ai, Yu Zeng, Wang-Jun Liao

**Affiliations:** 1 Department of Oncology, Nanfang Hospital, Southern Medical University, 510515 Guangzhou, Guangdong Province P.R. China; 2 Department of Hematology and Oncology, Shenzhen University General Hospital, 518055 Shenzhen, Guangdong Province P.R. China; 3 Department of General Surgery, Shenzhen University General Hospital, 518055 Shenzhen, Guangdong Province P.R. China; 4 Scientific research section, Shenzhen University General Hospital, 518055 Shenzhen, Guangdong Province P.R. China

**Keywords:** Colon cancer, Rectal cancer

## Abstract

Colorectal cancer (CRC) is the third most frequently diagnosed cancer with unfavorable clinical outcomes worldwide. circFNDC3B plays as a tumor suppressor in CRC, however, the mechanism of circFNDC3B in CRC remains ambiguous. The stem-like properties of CRC cells were detected by the evaluation of stemness markers, sphere formation assay and flow cytometry. qRT-PCR, FISH, IHC, and western blotting assessed the expression and localization of circFNDC3B, RNF41, ASB6, and stemness markers in CRC. The metastatic capabilities of CRC cells were examined by wound healing and Transwell assays, as well as in vivo liver metastasis model. Bioinformatics analysis, RNA immunoprecipitation (RIP), RNA pull-down assay and co-IP were used to detect the associations among circFNDC3B, FXR2, RNF41, and ASB6. Downregulated circFNDC3B was associated with unfavorite survival in CRC patients, and circFNDC3B overexpression suppressed CRC stemness and metastasis. Mechanistically, studies revealed that YTHDC1 facilitated cytoplasmic translocation of m^6^A-modified circFNDC3B, and circFNDC3B enhanced RNF41 mRNA stability and expression via binding to FXR2. circFNDC3B promoted ASB6 degradation through RNF41-mediated ubiquitination. Functional studies showed that silencing of RNF41 counteracted circFNDC3B-suppressed CRC stemness and metastasis, and ASB6 overexpression reversed circFNDC3B- or RNF41-mediated regulation of CRC stemness and metastasis. Elevated ASB6 was positively correlated with unfavorite survival in CRC patients. In vivo experiments further showed that circFNDC3B or RNF41 overexpression repressed tumor growth, stemness and liver metastasis via modulating ASB6. Taken together, m^6^A-modified circFNDC3B inhibited CRC stemness and metastasis via RNF41-dependent ASB6 degradation. These findings provide novel insights and important clues for targeted therapeutic strategies of CRC.

## Introduction

Colorectal cancer (CRC) is the 3rd most diagnosed cancer globally [1]. In recent years, the incidence of CRC rises among young and middle-aged adults [1, 2]. In the United States, there are ~106,180 estimated new cases and over 52,000 estimated CRC-related deaths in 2022 [1]. Despite advances in diagnostic approaches and therapies, the prognosis and overall survival (OS) of CRC remain poor mainly due to chemoresistance and metastasis [3]. It is of great clinical significance to elucidate the mechanism underlying key signaling, thereby improving patient outcomes.

N^6^-methyladenosine (m^6^A) is ubiquitous in mRNA and non-coding RNA (ncRNA), and it has emerged as a pivotal player in ncRNA metabolism [4, 5]. Accumulating evidence has revealed the association between m^6^A in ncRNA and cancer, and m^6^A modification in ncRNA is required for proliferation, metastasis and stemness-like properties of cancer cells [5, 6]. circular RNA (circRNA) are a group of single-stranded RNA with closed loops and devoid of the 3’ poly(A) tails or 5’ caps [7]. In recent years, circRNAs have attracted growing attention due to its regulatory role in various human diseases [8, 9]. circFNDC3B, also known as hsa_circ_0006156, is derived from back-spliced exons 5 and 6 of the FNDC3B gene. It functions as an important player in various cancers, including bladder cancer, gastric cancer and CRC [10–12]. Notably, downregulated circFNDC3B has been observed in CRC. circFNDC3B and circFNDC3B-enriched exosomes suppress CRC cell growth, metastasis and angiogenesis via miR-937-5p/TIMP3 axis [12]. Interestingly, the sites of m^6^A modification in circFNDC3B were predicted by SRAMP database, indicating the significance of m^6^A-modified circFNDC3B in CRC.

Ring finger protein 41 (RNF41), also known as Nrdp1, is an E3 ubiquitin ligase which responsible for ErbB3 and ErbB4 degradation [13, 14]. It is well studies and important for the progression of several cancers, including prostate cancer, glioma, breast cancer and CRC [15–18]. For instance, RNF41-mediated ErbB3 degradation inhibits the growth and motility of breast cancer cells [15]. In glioblastoma, RNF41 mediates the ubiquitination of Dvl2, leading to enhanced migration and invasion in glioblastoma [19]. More importantly, low expression of RNF41 has been observed in CRC tissues and cells, and RNF41 plays an indispensable role in reducing KITENIN-bound Dvl2 [18]. The upstream regulatory axis of RNF41 and its biological function merit in-depth investigation. Bioinformatics analysis based on RPISeq database (http://pridb.gdcb.iastate.edu/RPISeq/) revealed putative interactions among circFNDC3B, RNF41 and the RNA binding protein (RBP) FXR2. Intriguingly, FXR2 is highly expressed in CRC, and regulates gene expression by stabilizing the target mRNA [20, 21], indicating that FXR2 might act as a mediator between circFNDC3B and RNF41 in CRC cells.

Analysis based on BioGRID database (https://thebiogrid.org) predicted that RNF41 might be an E3 ubiquitin ligase responsible for ASB6 degradation. ASB6 is originally identified as an APS-interacting protein which is implicated in the insulin signaling [22]. In oral squamous cell carcinoma (OSCC), the elevated ASB6 is correlated with adverse survival in OSCC patients [23]. Subsequent study has illustrated that ASB6 promotes stemness and metastasis by modulating ER stress [24]. UALCAN database (http://ualcan.path.uab.edu/analysis.html) has shown that ASB6 is markedly upregulated in CRC, and CRC patients with elevated ASB6 expression exhibit poor prognosis, indicating its potential role in regulating CRC stemness and metastasis. However, little is known of the role of ASB6 in CRC.

In this study, we demonstrated that YTHDC1 facilitated cytoplasmic export of m^6^A-modified circFNDC3B, thereby suppressing stem-like and metastatic properties of CRC cells. In addition, circFNDC3B stabilized RNF41 via binding to FXR2, and circFNDC3B promoted ubiquitin-mediated degradation of ASB6 via recruiting RNF41, thus suppressing CRC stemness and metastasis. Our data reveal an essential role of circFNDC3B in CRC, and indicate that targeting circFNDC3B/FXR2/RNF41/ASB6 signaling may be a promising avenue for CRC therapy.

## Materials and methods

### Clinical specimen

A cohort of 58 CRC tissues and their normal counterparts were collected from CRC patients at Shenzhen University General Hospital. Diagnoses were confirmed by two pathologists independently. Written consents were obtained from all patients. Patients who received chemo- or radiotherapy before surgery, presented other malignancies, with incomplete clinicopathologic data were excluded from this study. This study was approved by Ethics Committee of Shenzhen University General Hospital.

### Cell culture and treatment

CRC cell lines LoVo, SW480, HCT116, SW620, and HCT8 cells, and normal colon cell line FHC cells were from ATCC (Manassas, VA, USA). The cell lines were authenticated by STR DNA profiling analysis and tested for mycoplasma contamination. FHC cells were cultured in DMEM/F12 containing 10 ng/ml cholera toxin, 25 mM HEPES, 5 μg/ml insulin, 100 ng/ml hydrocortisone, 5 μg/ml transferrin, and 10% FBS (Gibco, Grand Island, NY, USA). All CRC cell lines were grown in RPMI1640 containing 10% FBS (Gibco) at 37 °C in 5% CO_2_. For MG132 (Sigma-Aldrich, St. Louis, MO, USA) treatment, LoVo and HCT116 cells were treated with 10 μM MG132 for 6 h.

### Cell transfection

The full-length of circFNDC3B, RNF41, or ASB6 was cloned into lentiviral vector (Genecreat, Wuhan, China) as described [25]. Scramble shRNA, sh-circFNDC3B, sh-YTHDC1, sh-RNF41, and sh-FXR2 were from Genecreat. LoVo and HCT116 cells were transfected using Lipofectamine 3000 reagent (Invitrogen), and stable clones were selected using 1 μg/ml puromycin.

### Subcellular fractionation

Subcellular fractions were prepared using PARIS Kit (Invitrogen). Briefly, cells were collected and lysed with cell fractionation buffer. The cytoplasmic lysates (supernatants) were collected after centrifugation, and the nuclear lysates were prepared with cell disruption buffer (pellets). Both lysates were then subject to RNA isolation and qRT-PCR analysis. GAPDH and U6 served as the cytoplasmic and nuclear markers, respectively.

### RNA Fluorescence in situ hybridization and immunofluorescence staining

The probes specific to circFNDC3B was synthesized by RiboBio (Guangzhou, China). CRC cells were fixed with 4% PFA and permeabilized with 0.5% Triton X-100. RNA Fluorescence in situ hybridization (FISH) was conducted using the FISH Kit (RiboBio). For co-staining, the slides were stained with antibody against FXR2 followed by AF594-conjugated secondary antibody incubation. The localization of circFNDC3B and FXR2 were detected using a confocal microscope (Beckman, Jena, Germany).

### Methylated RNA immunoprecipitation-qPCR

Total RNA was isolated using TRIzol reagent (Invitrogen). Methylated RNA immunoprecipitation (MeRIP) was conducted using Magna MeRIP m^6^A kit (Millipore, Billerica, MA, USA). Briefly, anti-m^6^A antibody was conjugated to Protein A/G beads, followed by the incubation with fragmented RNA. The enriched RNA was purified and analyzed by qRT-PCR analysis.

### Sphere formation assay

LoVo and HCT116 cells (1 × 10^3^ cells/well) were reseeded in six-well ultra-low attachment culture dishes, and grown in RPMI1640 containing 20 ng/ml b-FGF, 2% B-27 supplement, 20 ng/ml EGF, 0.4% BSA, and 1% N-2 supplement (Gibco). After 14 days, the spheroid colonies (>50 μm in diameter) were photographed and counted.

### Flow cytometry

LoVo and HCT116 cells were harvested and digested with Accutase (Invitrogen). Cells were resuspended in surface staining buffer containing anti-CD133 antibody (Invitrogen) at 4 °C. The stained cells were then analyzed on a flow cytometer (BD Biosciences, Franklin Lakes, NJ, USA).

### Wound healing assay

CRC cells were grown in serum-free medium as described [26]. The monolayer was scratched using a pipette tip and rinsed with PBS. After 24 h, the wound healing processes were photographed using a microscope (Beckman).

### Transwell invasion assay

CRC cells were seeded in the Matrigel (BD Biosciences)-coated upper Transwell chamber, and maintained in serum-free RPMI1640. The bottom chamber was filled with RPMI1640 complete medium. The invaded cells were fixed with 4% PFA and stained with crystal violet. The images were acquired using a microscope (Beckman).

### RNA pull-down assay

RNA pull-down assay was carried out using RNA Pull-down Assay Kit (Pierce, Rockford, IL, USA). Biotinylated circFNDC3B probe was conjugated to streptavidin magnetic beads, followed by the incubation with protein lysates. The enriched RNA-protein complex was analyzed by Western blotting. Random probe was used as a negative control.

### RNA immunoprecipitation assay

RNA immunoprecipitation (RIP) was carried out using Magna RIP kit (Millipore). Briefly, cells were lysed in RIP lysis buffer. Anti-FXR2 antibody (2 μg, ab168852, Abcam) or normal rabbit IgG (2 μg, 10500C, Invitrogen) conjugated beads were incubated with cell lysates at 4 °C overnight. The enriched circFNDC3B or RNF41 mRNA was purified and analyzed by qRT-PCR.

### Co-immunoprecipitation

Protein lysates were extracted using IP lysis buffer (Pierce). Anti-ASB6 antibody (2 μg, sc-515649, Santa Cruz Biotechnology, Santa Cruz, CA, USA) or normal mouse IgG (2 μg, 10400C, Invitrogen) was incubated with cell lysates at 4 °C overnight, followed by the incubation with Protein A/G agarose (Pierce). The eluted proteins were then analyzed by Western blotting. Total cell lysates were used as input, and normal mouse IgG served as a negative control. For the detection of ASB6 ubiquitination, transfected CRC cells were treated with 10 μM MG132 for 6 h. The immunoprecipitated ubiquitin was detected by Western blotting.

### Animal study

For all animal studies, male BALB/c nude mice (5-week-old, *n* = 6/group) were obtained from SJA Laboratory Animal Co., Ltd (Hunan, China). Mice were randomly divided into three groups. HCT116 cells (5 × 10^6^ cells/0.2 ml PBS) stably expressing circFNDC3B and RNF41 were inoculated into the right flank of each mouse. Every 4 days, tumor volumes were measured. Tumor volume = 1/2 (length × width^2^). After 32 days, the tumors were harvested and subjected to subsequent analysis. In vivo liver metastasis study was conducted as previous describe [27]. Stable transfected HCT116 cells (2 × 10^6^ cells) were injected into the tail vein of nude mice. After 32 days, mice were sacrificed. Liver tissues were harvested and stained with Hematoxylin & Eosin (H&E). The metastatic nodules were photographed and counted. The investigator was blinded to the group allocation during the experiment. All animal studies were approved by the Animal Ethics Committee of Shenzhen University General Hospital.

### Histological analysis

The tumors or liver tissues were fixed with 4% PFA and embedded in paraffin. The sections were subjected to deparaffinization and rehydration. The slides were then stained with H&E as previously described [28]. For immunohistochemistry (IHC), the sections were subjected to antigen retrieval and blocking. The sections were then incubated with anti-ASB6 (1:100, PA552077, Invitrogen), anti-Ki67 (1:200, ab15580, Abcam), or anti-CD133 antibody (1:200, ab216323, Abcam) at 4 °C overnight. The slides were then incubated with corresponding secondary antibody (Invitrogen). Signal was visualized using the DAB substrate (Pierce).

### RNA isolation and qRT-PCR

Total RNA was isolated using TRIzol (Invitrogen). RNA quantity and quality were assessed using NanoDrop (Thermo Fisher Scientific). cDNA synthesis and qRT-PCR were conducted using PrimeScript RT Reagent and SyBr Premix Ex Taq (TaKaRa, Dalian, China), respectively. GAPDH or U6 small nuclear RNA was used as an internal control. The target gene expression was calculated using 2 ^–ΔΔCT^ method. Primers used in this study was listed in Table 1. For mRNA stability assay, cells were incubated with Actinomycin D (5 μg/ml, Sigma-Aldrich) for 0, 4, 8, 12, and 16 h. Cells were incubated with RNase R (20 U/μL, Epicenter, WI, USA) for 30 min. The expression of circFNDC3B or RNF41 was examined by qRT-PCR.

**Table 1:** Primers used for qRT-PCR analysis.

Genes	Primer sequences (5′-3′)
circFNDC3B	F: 5′-GCAAGAAGCAGCCCAAAGTC-3′
	R: 5′-CATGGCTGAGGGGTAGCTTG-3′
OCT4	F: 5′-CTTGAATCCCGAATGGAAAGGG-3′
	R: 5′-GTGTATATCCCAGGGTGATCCTC-3′
Nanog	F: 5′-GTCCCAAAGGCAAACAACCC-3′
	R: 5′-ATCCCTGCGTCACACCATTG-3′
SOX2	F: 5′-GCCCTGCAGTACAACTCCAT-3′
	R: 5′-GACTTGACCACCGAACCCAT-3′
CD133	F: 5′-ATGCTCTCAGCTCTCCCGC-3′
	R: 5′-TTCTGTCTGAGGCTGGCTTG-3′
RNF41	F: 5′-AAAATGTGAGAAGGGAGCAGCA-3′
	R: 5′-CACAGTCGCTGAGGTGAGAC-3′
ASB6	F: 5′-GGAAAGCCCACTCTCCCTTTT-3′
	R: 5′-CTTGACGTCTGCCCCATGCT-3′
GAPDH	F: 5′-CCAGGTGGTCTCCTCTGA-3′
	R: 5′-GCTGTAGCCAAATCGTTGT-3′
U6	F: 5′-CTCGCTTCGGCAGCACA-3′
	R: 5′-AACGCTTCACGAATTTGCGT-3′

### Western blotting

Protein lysates were prepared in RIPA lysis buffer (Pierce) and quantified using Bradford reagent (Pierce). Equal amount of protein lysates was separated by gel electrophoresis. Proteins were then transferred onto PVDF membrane (Pierce). After 5% non-fat milk blocking, the blots were subjected to the incubation with anti-YTHDC1 (1:500, ab122340, Abcam), anti-FXR2 (1:2000, ab168852, Abcam), ubiquitin (1:1000, ab140601, Abcam), anti-RNF41 (1:1000, ab151231, Abcam), anti-ASB6 (1:1000, sc-515649, Santa Cruz), OCT4 (1:1000, ab181557, Abcam), Nanog (1:3000, ab109250, Abcam), SOX2 (1:1000, ab92494, Abcam), CD133 (1:2000, ab222782, Abcam), or anti-GAPDH antibody (1:1000, ab8245, Abcam) at 4 °C overnight. This is followed by the incubation with corresponding secondary antibody (Invitrogen). An ECL kit (Pierce) was employed to visualize the signals.

### Statistical analysis

All experiments were performed for at least three times. Statistical tests were performed using GraphPad Prism (GraphPad, San Diego, CA, USA). All data were in a normal distribution, and variance was similar between the groups that are being statistically compared. Survival curve was calculated using the Kaplan-Meier analysis. For multiple comparison, differences were analyzed by one-way ANOVA with Turkey post hoc test. For two-group comparison, statistical test was performed using Student’s *t* test. *P* < 0.05 was considered to be statistically significant.

## Results

### circFNDC3B is downregulated in CRC, and low circFNDC3B expression is associated with unfavorable overall survival in CRC patients

**Fig. 1:**
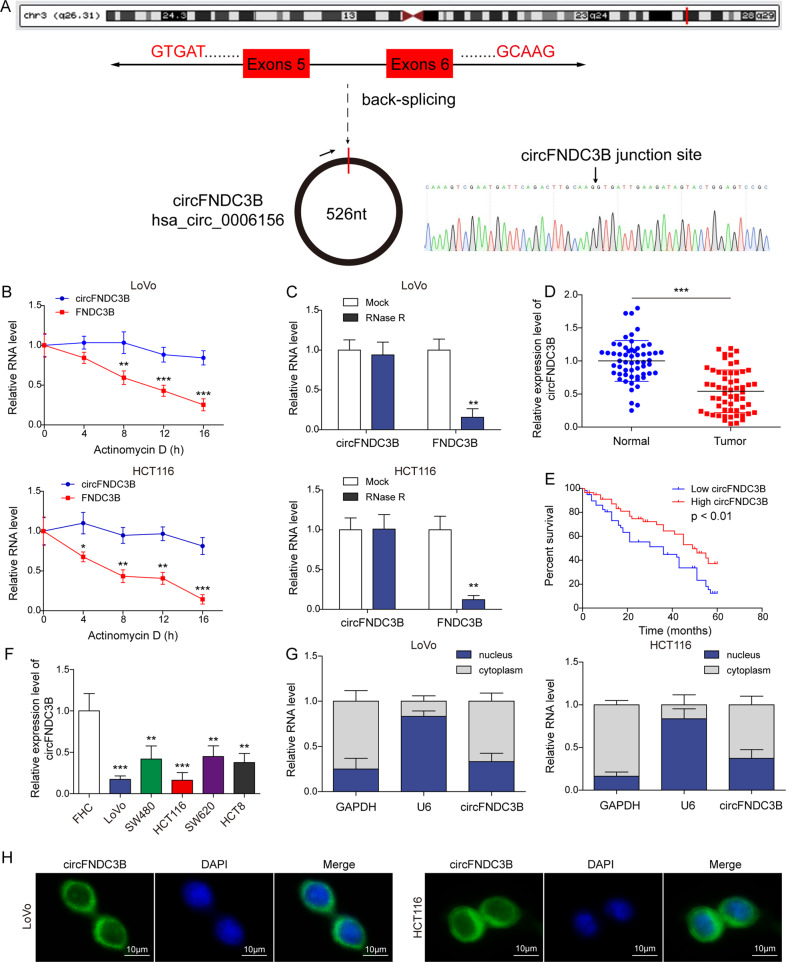
circFNDC3B is downregulated in CRC tissues and cells, and low circFNDC3B expression is associated with unfavorable overall survival in CRC patients. **A** Schematic drawing illustrated the formation of circFNDC3B. **B**, **C** The expression level of circFNDC3B was detected by qRT-PCR in LoVo and HCT116 cells treated with Actinomycin D or RNase R. **D** The circFNDC3B level in CRC tissues was detected by qRT-PCR. **E** The correlation between circFNDC3B level and OS of CRC patients was analyzed by Kaplan–Meier method. **F** The circFNDC3B level in CRC cells was detected by qRT-PCR. **G** The circFNDC3B level in LoVo and HCT116 cells was detected by subcellular fractionation. **H** The subcellular localization of circFNDC3B was detected by RNA FISH in LoVo and HCT116 cells. Scale bar, 10 μm; Green, circFNDC3B; Blue, DAPI. **P < 0.01, ***P < 0.001.

To unravel the function of circFNDC3B in CRC, we examined its expression in CRC tissues. circFNDC3B, also known as hsa_circ_0006156, is originated from exons 5 and 6 of the FNDC3B gene with 526 nt in length (Fig. 1A). circFNDC3B degraded time-dependently upon Actinomycin D treatment, and the degradation of circFNDC3B was much slower than that of linear FNDC3B in the presence of Actinomycin D or RNase R (Fig. 1B, C). circFNDC3B was dramatically decreased in CRC tissues, compared with their normal counterparts (Fig. 1D). In addition, the lower circFNDC3B level was positively correlated with TNM staging and lymph node metastasis of CRC patients, and no correlation with patient gender, age, tumor size, location, and histological type was observed (Table 2). CRC patients with low expression of circFNDC3B was correlated with unfavorable OS (Fig. 1E), indicating the clinical significance of circFNDC3B in CRC. Consistently, qRT-PCR showed that circFNDC3B levels in CRC cells were significantly lower than that in normal colon cell line FHC cells, and circFNDC3B was predominantly expressed in the cytoplasm of LoVo and HCT116 cells (Fig. 1F, G). RNA FISH further confirmed the cytoplasmic localization of circFNDC3B in LoVo and HCT116 cells (Fig. 1H). These findings suggest that the reduced circFNDC3B expression is correlated with unfavorite OS in CRC patients.

**Table 2:** Association between circFNDC3B expression levels and clinicopathological characteristics of CRC patients.

Clinical parameters	Cases (*n)*	circFNDC3B expression		*P*-value (*p < 0.05)
		High (*n*)	Low (*n*)	
Gender				0.592
Male	35	19	16	–
Female	23	10	13	–
Age				0.792
<60	26	12	14	–
≥60	32	17	15	–
Tumor size (cm)				0.065
<5	28	18	10	–
≥5	30	11	19	–
Location				0.292
Rectum	27	16	11	–
Colon	31	13	18	–
TNM stage				0.033
I–II	25	17	8	–
III–IV	33	12	21	–
Histological type				0.182
Differentiated	24	15	9	–
Undifferentiated	34	14	20	–
Lymph node metastasis				0.017
Yes	26	8	18	–
No	32	21	11	–

*CRC* colorectal cancer, *TNM* tumor nodes metastasis.

### circFNDC3B suppresses CRC stemness and metastasis

**Fig. 2:**
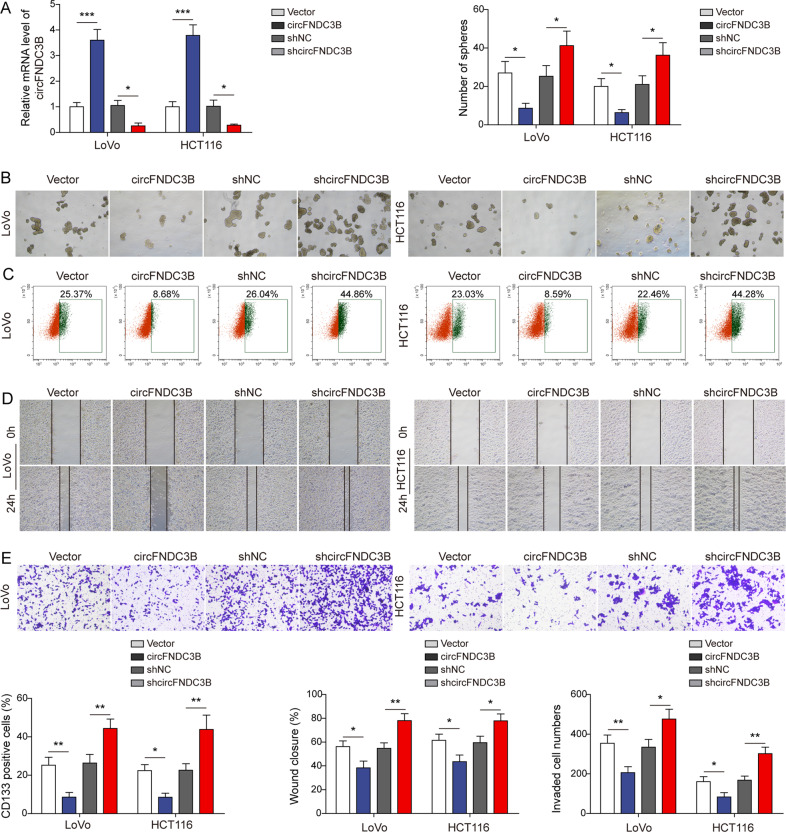
circFNDC3B suppresses CRC stemness and metastasis. **A** The circFNDC3B level in transfected CRC cells was detected by qRT-PCR. **B** Representative sphere images of transfected CRC cells. **C** CD133^+^ cells were analyzed by flow cytometry with quantitative analysis in CRC cells transfected with circFNDC3B overexpression vector or sh-circFNDC3B. **D** Cell migration was monitored by wound healing assay with quantitative analysis in CRC cells transfected with circFNDC3B overexpression vector or sh-circFNDC3B. **E** Cell invasion was measured by Transwell invasion assay with quantitative analysis in CRC cells transfected with circFNDC3B overexpression vector or sh-circFNDC3B. *P < 0.05, **P < 0.01, ***P < 0.001.

Functional assays were next conducted to investigate the effects of circFNDC3B on CRC stemness and metastasis. LoVo and HCT116 cells with relatively low circFNDC3B expression were selected for subsequent experiments. As shown in Fig. 2A, transfection of circFNDC3B overexpression construct or sh-circFNDC3B successfully increased or decreased circFNDC3B level in LoVo and HCT116 cells, respectively. circFNDC3B overexpression inhibited sphere formation, whereas the number of spheres formed was increased in circFNDC3B-knockdown CRC cells (Fig. 2B). In accordance with this result, flow cytometry showed that circFNDC3B negatively regulated the proportion of CD133-positive cells, while silencing of circFNDC3B exerted an opposite effect (Fig. 2C). Moreover, overexpression of circFNDC3B impaired the migratory and invasive capacities of CRC cells, while the increased migration and invasion were observed in circFNDC3B-knockdown cells (Fig. 2D, E). These data indicate that circFNDC3B may act as a key player in CRC stemness and metastasis.

### YTHDC1 promotes cytoplasmic translocation of m^6^A-modified circFNDC3B

**Fig. 3:**
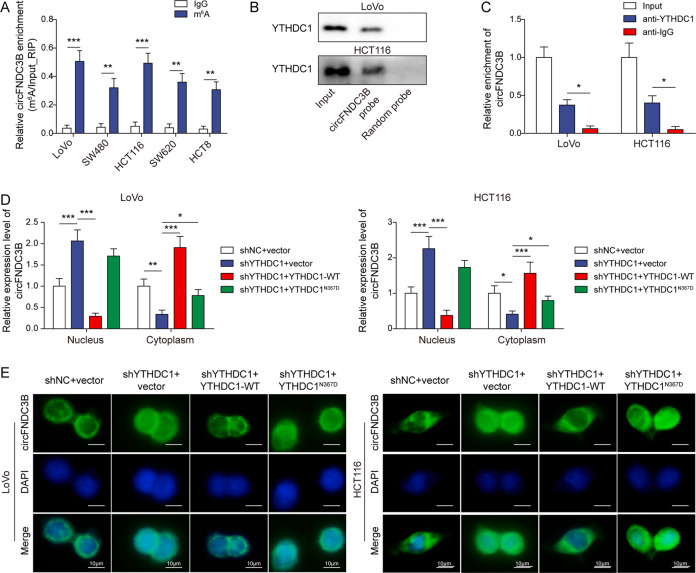
YTHDC1 promotes cytoplasmic translocation of m^6^A modified circFNDC3B. **A** m^6^A modification of circFNDC3B was detected by MeRIP-qPCR. **B** The interaction between biotinylated circFNDC3B and YTHDC1 was verified by RNA pull-down assay. Random probe acted as a negative control. **C** The interaction between circFNDC3B and YTHDC1 was detected by RIP assay. Normal IgG served as a negative control. **D** The circFNDC3B level in CRC cells was detected by subcellular fractionation and qRT-PCR. **E** The subcellular localization of circFNDC3B was detected by RNA FISH. Scale bar, 10 μm; Green, circFNDC3B; Blue, DAPI. **P < 0.01, ***P < 0.001.

In accordance with the bioinformatics analysis, m^6^A modification of circFNDC3B was detected in CRC cells by MeRIP-qPCR (Fig. 3A). A recent study has illustrated that YTH domain-containing protein 1 (YTHDC1) facilitates cytoplasmic transportation of circNSUN2 in an m^6^A methylation-dependent manner, thus stabilizing HMGA2 to promote liver metastasis in CRC [29], raising the possibility that YTHDC1 might be implicated in the cytoplasmic export of circFNDC3B in CRC cells. RNA pull-down assay revealed that biotinylated circFNDC3B successfully enriched YTHDC1 in both CRC cells (Fig. 3B). On the other hand, RIP assay further confirmed that the antibody against YTHDC1 successfully immunoprecipitated circFNDC3B in CRC cells (Fig. 3C), indicating the direct association between circFNDC3B and YTHDC1. We next tested the effct of YTHDC1 on the subcellular localization of circFNDC3B in CRC cells. As shown in Fig. 3D, E, qRT-PCR and RNA FISH showed that silencing of YTHDC1 inhibited the translocation of circFNDC3B from the nucleus to the cytoplasm, and co-transfection of wild-type YTHDC1 (YTHDC1-WT) reversed this effect. It is worth noting that overexpression of mutated YTHDC1 (YTHDC1^N367D^) had no remarkable effect on the nuclear localization of circFNDC3B in CRC cells, compared with shYTHDC1+vector group (Fig. 3D, E). The successful subcellular fractionation was verified by qRT-PCR, and knockdown or overexpression of YTHDC1 had no effect on GAPDH and U6 expression in cytoplasm and nucleus (Fig. S1). These findings suggest that YTHDC1 facilitates cytoplasmic translocation of m^6^A modified circFNDC3B.

### CircFNDC3B increases RNF41 mRNA stability and expression via binding to FXR2

**Fig. 4:**
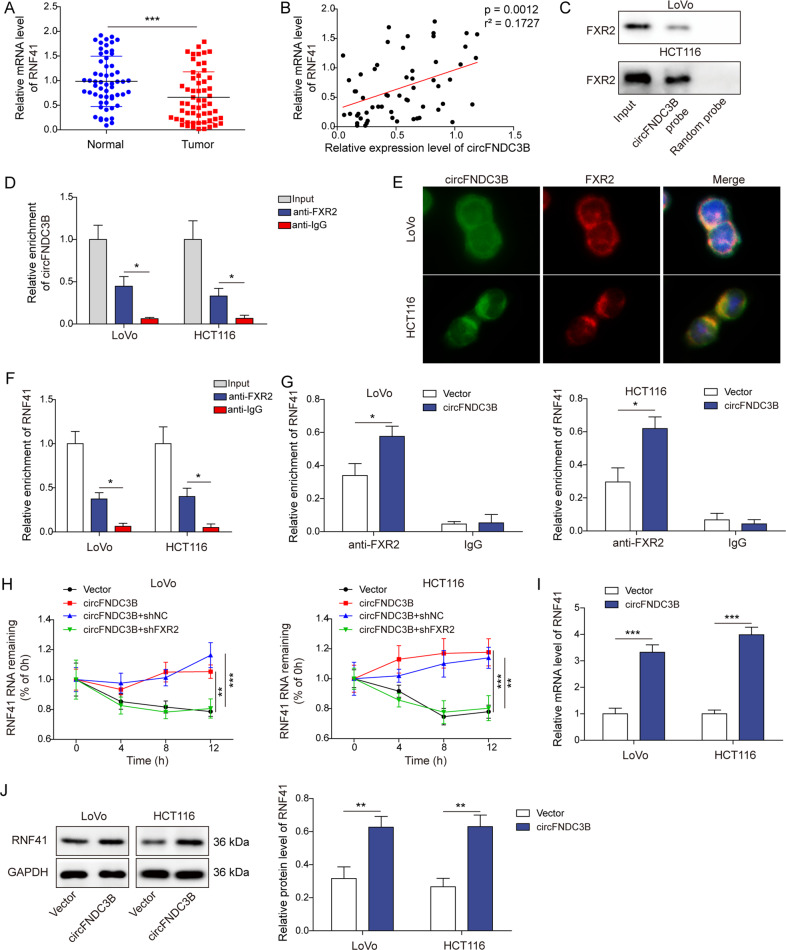
circFNDC3B increases RNF41 mRNA stability and expression via binding to FXR2. **A** The RNF41 level in CRC tissues was detected by qRT-PCR. **B** The correlation between circFNDC3B and RNF41 level in CRC tissues was analyzed by spearman correlation analysis. **C** The interaction between biotinylated circFNDC3B and FXR2 was detected by RNA pull-down assay. Random probe acted as a negative control. **D** The interaction between circFNDC3B and FXR2 was detected by RIP assay. Normal IgG served as a negative control. **E** The co-localization of circFNDC3B and FXR2 was detected by RNA FISH and immunofluorescence (IF). Scale bar, 10 μm; Green, circFNDC3B; Red, FXR2; Blue, DAPI. **F**, **G** The interaction between RNF41 and FXR2 was detected by RIP assay. Normal IgG served as a negative control. **H** The mRNA stability of RNF41 was determined by RNA stability assay. **I**, **J** The mRNA and protein levels of RNF41 in transfected CRC cells were detected by qRT-PCR and Western blotting. *P < 0.05, **P < 0.01, ***P < 0.001.

As presented in Fig. 4A, RNF41 was downregulated in CRC tissues, compared with their normal counterparts. circFNDC3B positively correlated with RNF41 in CRC tissues (Fig. 4B). To test direct interaction between circFNDC3B and FXR2, RNA pull-down assay was conducted. Biotinylated circFNDC3B successfully pulled down FXR2 in CRC cells (Fig. 4C). Consistently, RIP assay confirmed that anti-FXR2 antibody immunoprecipitated circFNDC3B in both LoVo and HCT116 cells (Fig. 4D). RNA FISH coupled with immunofluorescence staining revealed that circFNDC3B co-localized with FXR2 in LoVo and HCT116 cells (Fig. 4E). Intriguingly, RIP assay also showed a direct association between FXR2 and RNF41 mRNA (Fig. 4F), and overexpression of circFNDC3B potentiated this interaction in CRC cells (Fig. 4G). In addition, circFNDC3B overexpression enhanced the mRNA stability of RNF41 in the presence of transcription inhibitor ActD, whereas FXR2 knockdown reversed circFNDC3B-mediated RNF41 mRNA stabilization (Fig. 4H). qRT-PCR and Western blotting further revealed that circFNDC3B overexpression increased RNF41 expression (Fig. 4I, J). These data indicate that circFNDC3B directly binds to FXR2 and increases RNF41 mRNA stability and expression in CRC cells.

### Knockdown of RNF41 counteracts circFNDC3B-suppressed CRC stemness and metastasis

**Fig. 5:**
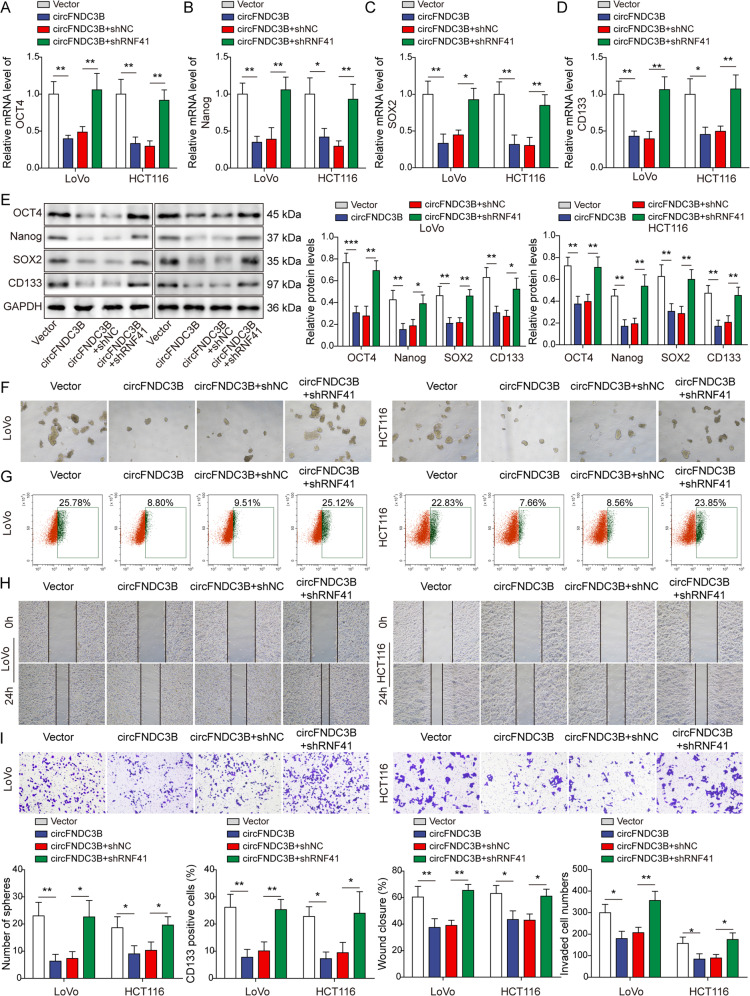
Knockdown of RNF41 counteracts circFNDC3B-suppressed CRC stemness and metastasis. **A**–**E** The expression of stemness markers were detected by qRT-PCR and Western blotting. **F** Representative sphere images of transfected CRC cells with quantitative analysis. **G** CD133^+^ cells were analyzed by flow cytometry with quantitative analysis in transfected CRC cells. **H** Cell migration was monitored by wound healing assay with quantitative analysis in transfected CRC cells. **I** Cell invasion was measured by Transwell invasion assay with quantitative analysis. *P < 0.05, **P < 0.01.

We next sought to study the functions of RNF41 in circFNDC3B-mediated regulation of CRC stemness and metastasis. As shown in Fig. 5A–E, circFNDC3B-decreased expression of stemness markers OCT4, Nanog, SOX2 and CD133 were rebound in circFNDC3B + shRNF41 group as detected by qRT-PCR and Western blotting. As expected, sphere formation assay showed that circFNDC3B-impaired sphere formation was rescued by shRNF41 (Fig. 5F). circFNDC3B-decreased proportion of CD133-positive cells was increased in circFNDC3B + shRNF41 group (Fig. 5G). Additionally, wound healing and Transwell invasion assays revealed that circFNDC3B-inhibted migration and invasion were also attenuated by shRNF41 (Fig. 5H, I). These data suggest that RNF41 functions as a downstream effector in circFNDC3B-suppressed CRC stemness and metastasis.

### circFNDC3B promotes ASB6 degradation through RNF41-mediated ubiquitination

**Fig. 6:**
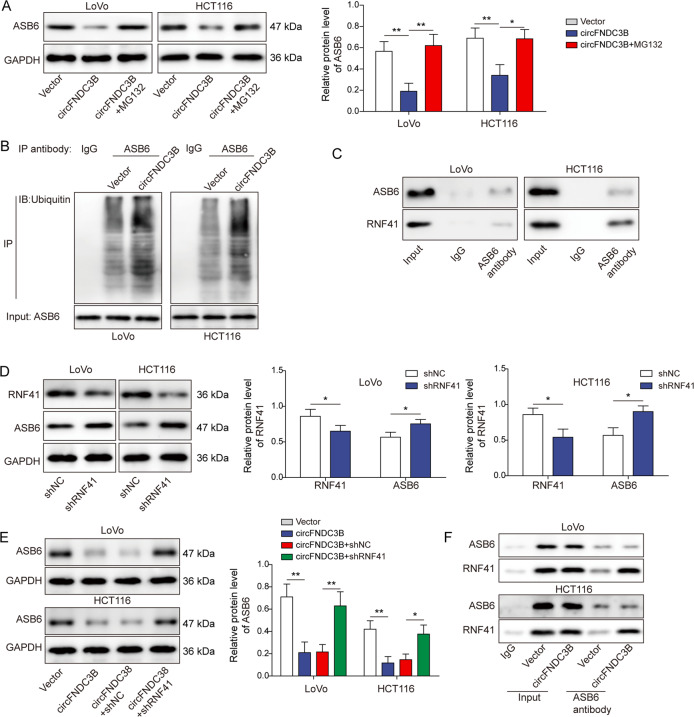
circFNDC3B promotes ASB6 degradation through RNF41-mediated ubiquitination. **A** The protein level of ASB6 was determined by western blotting with quantitative analysis. **B** The ubiquitination of ASB6 was assessed by co-IP in transfected CRC cells. **C** The interaction between ASB6 and RNF41 was detected by Co-IP. Normal IgG served as a negative control. **D** The protein levels of RNF41 and ASB6 were determined by western blotting with quantitative analysis in transfected CRC cells. **E** The protein level of ASB6 was determined by western blotting with quantitative analysis in transfected CRC cells. **F** The interaction between ASB6 and RNF41 in circFNDC3B-overexpressing cells was detected by Co-IP. *P < 0.05, **P < 0.01.

Interestingly, circFNDC3B-decreased ASB6 expression was blocked by the proteasome inhibitor MG132 (Fig. 6A), indicating that circFNDC3B might promote ASB6 degradation via ubiquitin-proteasome pathway. Co-immunoprecipitation (Co-IP) further showed that overexpression of circFNDC3B promoted the ubiquitination of ASB6, compared with vector alone group (Fig. 6B). In order to test whether RNF41 was an E3 ubiquitin ligase responsible for circFNDC3B-mediated degradation of ASB6, a series of mechanistic experiments were carried out. A direct association between ASB6 and RNF41 was detected by Co-IP (Fig. 6C). Silencing of RNF41 caused a marked reduction of RNF41, but increased ASB6 expression in LoVo and HCT116 cells (Fig. 6D). Additionally, silencing of RNF41 reversed circFNDC3B-decreased ABS6 in CRC cells (Fig. 6E), indicating that RNF41 plays a critical role in circFNDC3B-mediated degradation of ASB6. Moreover, Co-IP revealed the enhanced the association between ASB6 and RNF41 by circFNDC3B overexpression (Fig. 6F). These findings indicate that circFNDC3B promotes ASB6 degradation via recruiting E3 ubiquitin ligase RNF41.

### ASB6 is elevated in CRC and associated with unfavorite OS in CRC patients

**Fig. 7:**
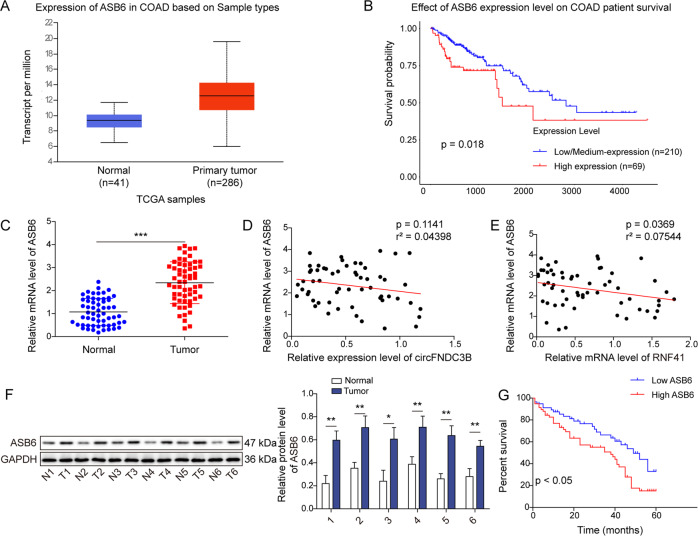
ASB6 is elevated in CRC and associated with poor OS in CRC patients. **A**, **B** Differential expression of ASB6 and survival analysis in CRC based on UALCAN database. **C** The mRNA level of ASB6 in CRC tissues was detected by qRT-PCR. **D**, **E** The correlation among circFNDC3B, RNF41 and ASB6 in CRC tissues was analyzed by spearman correlation analysis. **F** The protein level of ASB6 in CRC tissues was detected by western blotting. **G** The correlation between ASB6 level and OS of CRC patients was analyzed by Kaplan–Meier method. *P < 0.05, **P < 0.01, ***P < 0.001.

To investigate the role of ASB6 in CRC, we next evaluated ASB6 expression in CRC tissues. UALCAN data analysis revealed that ASB6 was remarkably upregulated in colorectal adenocarcinoma (COAD), and COAD patients with high ASB6 expression exhibited unfavorable OS (Fig. 7A, B). In consistent with these findings, qRT-PCR suggested that ASB6 was markedly elevated in CRC tissues, compared with adjacent normal tissues (Fig. 7C). There was no significant correlation between circFNDC3B and ASB6 expression in CRC tissues (Fig. 7D), and RNF41 negatively correlated with ASB6 in CRC tissues (Fig. 7E). Additionally, Western blotting suggested that the protein level of ASB6 was markedly elevated in CRC tissues (Fig. 7F). Kaplan–Meier analysis indicated that high ASB6 expression was associated with unfavorite OS in patients with CRC (Fig. 7G). Together, these finding indicate that high expression of ASB6 is associated with unfavorite prognosis in CRC patients.

### Overexpression of ASB6 reverses circFNDC3B or RNF41-mediated regulation of CRC stemness and metastasis

**Fig. 8:**
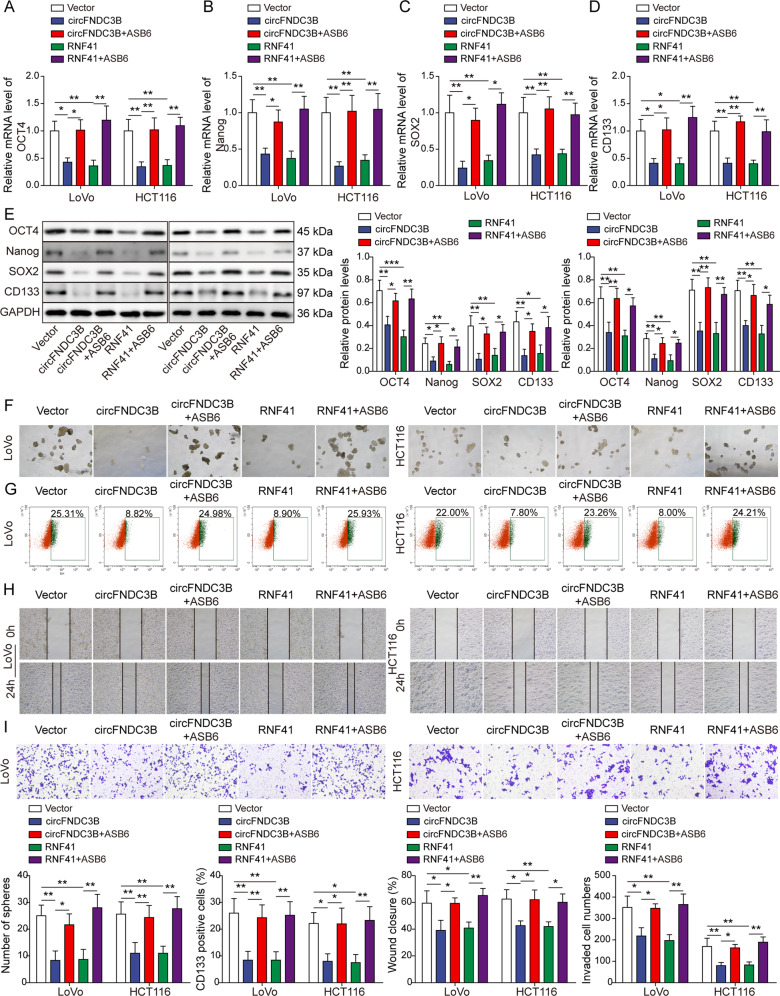
Overexpression of ASB6 reverses circFNDC3B or RNF41-mediated regulation of CRC stemness and metastasis. **A**–**E** The expression of stemness markers were detected by qRT-PCR and western blotting. **F** Representative sphere images of transfected CRC cells with quantitative analysis. **G** CD133^+^ cells were analyzed by flow cytometry with quantitative analysis in transfected CRC cells. **H** Cell migration was monitored by wound healing assay with quantitative analysis in transfected CRC cells. **I** Cell invasion was measured by Transwell invasion assay with quantitative analysis. *P < 0.05, **P < 0.01.

Rescue experiments were next conducted to delineate the functions of ASB6 in circFNDC3B- or RNF41-mediated regulation of CRC stemness and metastasis. As presented in Fig. 8A–E, circFNDC3B or RNF41 decreased the expression of OCT4, Nanog, SOX2, and CD133, while ASB6 overexpression resulted in a rebound of these stemness markers in CRC cells. In addition, circFNDC3B- or RNF41-impaired sphere formation was rescued by ASB6 overexpression (Fig. 8F). Flow cytometry further revealed that circFNDC3B- or RNF41-reduced population of CD133-positive cells was counteracted by ASB6 (Fig. 8G). For the metastatic properties, wound healing and Transwell invasion assays revealed that circFNDC3B- or RNF41-impaired migratory and invasive capacities of CRC cells were rescued in circFNDC3B + ASB6 and RNF41 + ASB6 groups (Fig. 8H, I). These data suggest that ASB6 serves as a key downstream molecule in circFNDC3B/RNF41-regulated CRC stemness and metastasis.

### Overexpression of circFNDC3B or RNF41 suppresses tumor growth, stemness, and liver metastasis via modulating ASB6 in vivo

**Fig. 9:**
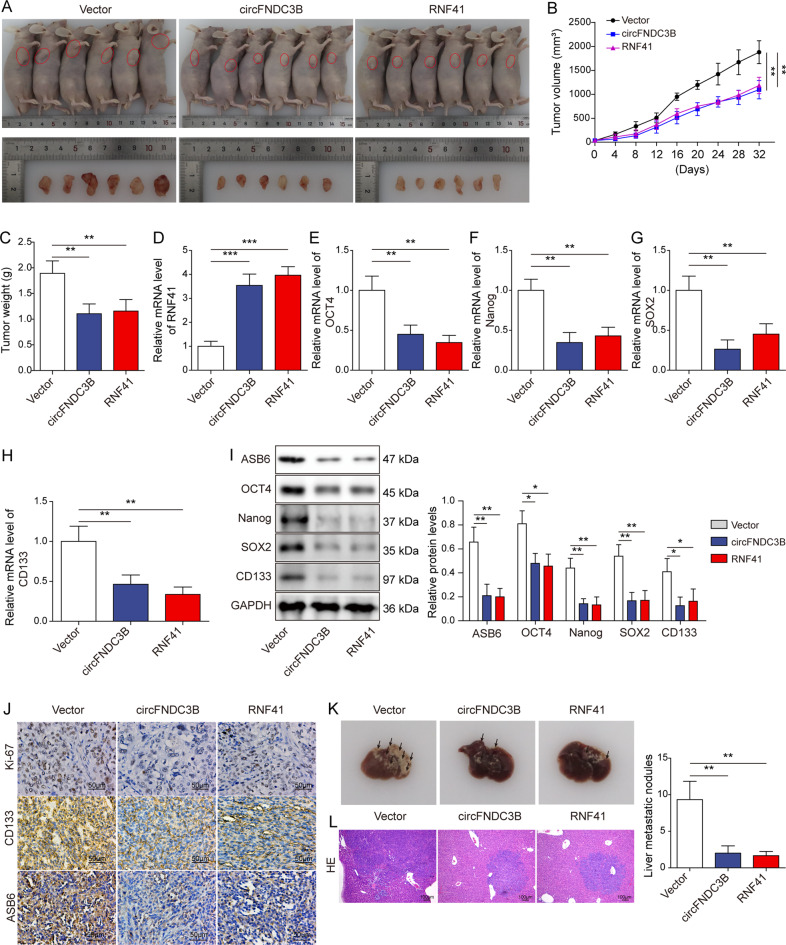
Overexpression of circFNDC3B or RNF41 suppresses tumor growth, stemness and liver metastasis via modulating ASB6 in vivo. **A** Photographs of xenograft tumors. **B** Quantitative analysis of tumor volume. **C** Quantitative analysis of tumor weight. **D**–**H** The mRNA levels of RNF41, OCT4, Nanog, SOX2, and CD133 in xenograft tumors were detected by qRT-PCR. **I** The protein levels of ASB6, OCT4, Nanog, SOX2, and CD133 were detected by western blotting with quantitative analysis. **J** The immunoreactivities of Ki-67, CD133, and ASB6 in xenograft tumors were detected by IHC analysis. **K** Representative photographs of liver tissues derived from in vivo liver metastasis mice model. **L** The histological changes of liver tissues were detected by H&E staining. *P < 0.05, **P < 0.01, ***P < 0.001.

Animal studies were next performed to validate the in vitro findings. Xenograft study revealed that overexpression of circFNDC3B or RNF41 remarkably decreased tumor volumes and weight (Fig. 9A–C), accompanied with increased RNF41 level in xenograft tumors (Fig. 9D). In line with the in vitro findings, the stemness-related markers OCT4, Nanog, SOX2, and CD133 were decreased by circFNDC3B or RNF41 overexpression in vivo (Fig. 9E–I). More importantly, ASB6 was also downregulated in circFNDC3B-or RNF41-overexpressing xenograft tumors (Fig. 9I). IHC analysis further confirmed the circFNDC3B- or RNF41-mediated downregulation of ASB6 in tumor tissues, and the proliferation marker Ki-67 and stemness marker CD133 were also reduced by circFNDC3B or RNF41 overexpression in vivo (Fig. 9J). In vivo liver metastasis study revealed that circFNDC3B or RNF41 overexpression inhibited liver metastasis in which the number of metastatic nodules was decreased in circFNDC3B- or RNF41-overexpressing groups (Fig. 9K, L). Collectively, these data indicate that circFNDC3B or RNF41 overexpression suppresses tumor growth, stemness and liver metastasis via modulating ASB6 in vivo.

## Discussion

In recent years, rising incidence of CRC among younger patients have been reported worldwide [30]. Metastasis, especially liver metastasis, and recurrence lead to poor prognosis and high mortality of CRC [30, 31]. Epigenetic modifications have emerged as important regulators in cancer development and progression, and the epigenetic-targeted therapy is considered promising for cancer treatment [32, 33]. In this study, we reported that m^6^A-modified circFNDC3B stabilized and recruited RNF41 in a FXR2-dependent manner, thereby promoting ubiquitin-mediated degradation of ASB6 and suppressing CRC stemness and metastasis. These findings provided an in-depth understanding for epigenetic-targeted therapy of CRC.

Great progress in understanding and unraveling the function of circRNAs has been accomplished in recent years. Aberrant expression of circRNAs has been found in CRC, and they serve as valuable diagnostic and prognostic markers [34]. In particular, circRNAs play key roles in proliferation, apoptosis, metastasis and drug resistance of CRC [35]. Mechanistically, circRNAs act as sponges of microRNA (miRNA) and encode for proteins [34, 36]. Given the key roles of m^6^A modification in circRNA metabolism, the function of m^6^A-modified circRNA has attracted increasing attention in recent years [37]. In CRC, low circFNDC3B level has been observed, and circFNDC3B/miR-937-5p/TIMP3 axis contributes to cancer progression [12]. A recent study has also reported that circFNDC3B-218aa, a newly identified protein encoded by circFNDC3B, suppresses CRC progression and epithelial-mesenchymal transition (EMT) by inhibiting Snail-FBP1 axis [38]. However, little research focused on the regulatory mechanism of m^6^A-modified circFNDC3B in CRC. In accordance with previous reports, our finding showed that downregulated circFNDC3B was associated with unfavorite prognosis of CRC patients. circFNDC3B inhibited stemness and metastasis in CRC cells, indicating its anti-oncogenic role in CRC.

m^6^A modification is originally identified as a predominant modification of mRNA, and its pivotal roles in ncRNA modification have emerged recently [4, 5]. YTHDC1, one of the most noted m^6^A reader, recognizes m^6^A modification on mRNAs or ncRNAs and mediates different biological processes, such as cytoplasmic export, RNA stabilization and decay [39]. Notably, YTHDC1 plays critical roles in different cancers [39, 40], and the elevation of YTHDC1 is observed in CRC cells and tissues [41]. Recent studies have demonstrated that YTHDC1 facilitates cytoplasmic delivery of circMET in renal cell carcinoma [42], as well as the cytoplasmic translocation of circHPS5 in hepatocellular carcinoma (HCC) in an m^6^A-dependent manner [43]. More importantly, YTHDC1 also recognizes m^6^A-modified circNSUN2 and facilitates its cytoplasmic transportation, thereby stabilizing HMGA2 mRNA to enhance CRC liver metastasis [29]. Similarly, our data showed that there was a direct interaction between YTHDC1 and circFNDC3B in CRC cells, and YTHDC1 was required for the cytoplasmic export of circFNDC3B in LoVo and HCT116 cells. Our findings extend the understanding regarding the significance of m^6^A modification in circRNAs.

FXR2 belongs to FXR family which is implicated in neuronal maturation, RNA metabolism and muscle development [44]. Mechanistic study has illustrated that FXR2 modulates gene expression by binding to the target mRNA [20]. For instance, FXR2 is required for the stabilization of GluA1 mRNA in neurons and Fxr2 knockout mice [45]. Consistently, we reported that FXR2 served as a key mediator between circFNDC3B and RNF41 in CRC cells. It is well-known that E1 ubiquitin-activating enzyme, E2 ubiquitin-activating enzyme and E3 ubiquitin ligase are enzyme cascades implicated in protein ubiquitination [46]. RNF41 is a well-known E3 ubiquitin ligase participate in ErbB3/ErbB4 degradation [13, 14]. Previous study has demonstrated that RNF41 is required for degradation of KITENIN-bound Dvl2, as well as generation of c-Jun by the EGF-KITENIN/ErbB4 complex in CRC [18]. In this study, RNF41 was identified as an E3 ubiquitin ligase responsible for circFNDC3B-promoted ASB6 degradation.

ASB6 acts as an adapter protein which participates in the activation of insulin signaling [22], however, little is known about its role in cancer. A recent study has illustrated that ASB6 regulates HCC proliferation and autophagy via promoting p62 ubiquitination and degradation [47]. Consistent with the analysis based on UALCAN database, we found that upregulated ASB6 was associated with adverse prognosis in CRC patients. Previous study has revealed that ASB6 enhances the stem-like and metastatic properties of OSCC cells via attenuating ER stress [24]. Similar with the pro-stemness role of ASB6 in OSCC, functional studies further confirmed that ASB6 acted as a downstream effector in circFNDC3B/RNF41-mediated regulation of CRC stemness and metastasis. Additionally, we found that circFNDC3B decreased ASB6 protein level, and the proteasome inhibitor MG132 led to a rebound of ASB6, indicating that circFNDC3B regulates ASB6 expression via ubiquitin-proteasome pathway. Our findings first demonstrated the role of RNF41/ASB6 axis in m^6^A modification of circFNDC3B in CRC.

In conclusion, we reported for the first time that m^6^A-modified circFNDC3B suppressed CRC stemness and metastasis via RNF41-dependent ASB6 degradation. Our findings broaden the understanding of m^6^A-modified circFNDC3B in CRC, and identified novel candidates for targeted therapy.

## Supplementary information

Supplementary Figure 1

Supplementary Figure legends

aj-checklist

## Data Availability

All data generated or analyzed during this study are included in this published article.
