# Supplementary figures and images for: m6A-modified circFNDC3B inhibits colorectal cancer stemness and metastasis via RNF41-dependent ASB6 degradation
Source: Cell Death Dis. 2022 Nov 29;13(11):1008. doi: 10.1038/s41419-022-05451-y (PMC9709059; doi:10.1038/s41419-022-05451-y)

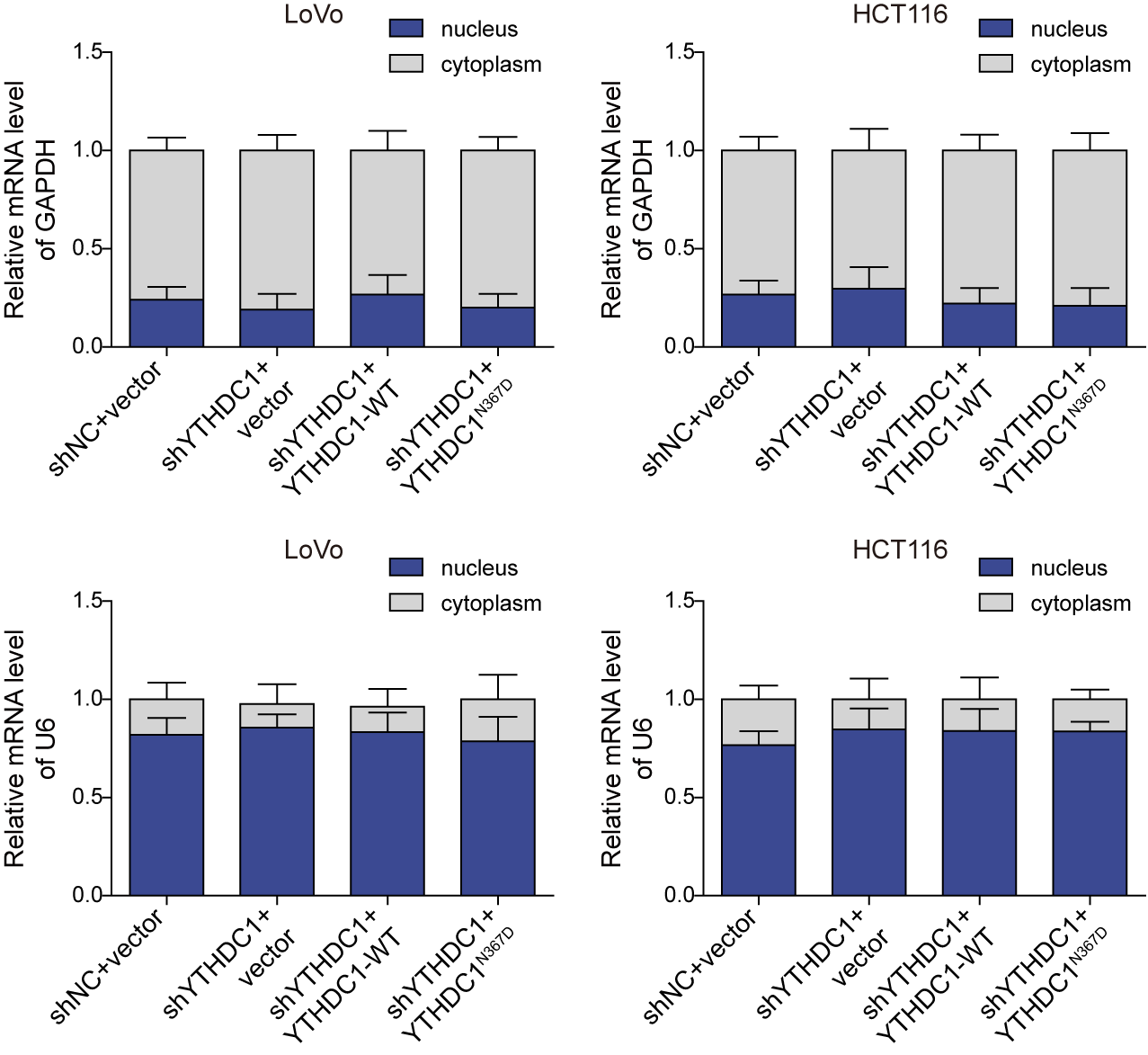

Supplement: Supplementary file 1 — Supplementary Figure 1 [file 41419_2022_5451_MOESM1_ESM.tif]
