# Supplementary material for: m6A-modified circFNDC3B inhibits colorectal cancer stemness and metastasis via RNF41-dependent ASB6 degradation
Source: Cell Death Dis. 2022 Nov 29;13(11):1008. doi: 10.1038/s41419-022-05451-y (PMC9709059; doi:10.1038/s41419-022-05451-y)
Supplement: Supplementary file 2 — Supplementary Figure legends [file 41419_2022_5451_MOESM2_ESM.docx]

**Figure S1. The successful subcellular fractionation was verified by qRT-PCR.** GAPDH and U6 acted as a cytoplasmic and a nuclear marker, respectively.
